# How to optimize culture media osmolality during Assisted Reproductive Technologies treatments

**DOI:** 10.5935/1518-0557.20210123

**Published:** 2023

**Authors:** Renata de Lima Bossi, Brenda Campos Villa Pinto, Marcos Aurelio Coelho Sampaio, Selmo Geber

**Affiliations:** 1 Centro de Medicina Reprodutiva - ORIGEN, Belo Horizonte, MG, Brazil

**Keywords:** osmolality, culture medium, incubator, oil, human assisted reproduction

## Abstract

**Objective:**

The objective of our study was to compare the osmolality in sequential and single step culture media, used for in vitro human embryo culture, covered with mineral oil and paraffin, in dry and humid incubators.

**Methods:**

We performed a prospective observational study. A total of 120 Petri dishes, with 960 droplets of culture media, were evaluated. Each dish was prepared with 4 droplets of single step medium and sequential medium. Sixty dishes were covered with mineral oil and 60 with paraffin oil. Half were incubated in a dry incubator and half in a humid. Osmolality was measured on days 1, 3, 5, 7. ANOVA test was performed for statistical analysis.

**Results:**

Osmolality results for single step and sequential medium, that were covered with both mineral and paraffin oil and placed in the dry incubator, significantly increased throughout the study time (D7>D5>D3). In the humid incubator, the results were similar for all periods. Osmolality was significantly lower in humid incubator, in all periods, when droplets were covered with both oils. When both culture media were placed in the humid incubator, no variation was detected, using both oils. However, when single step medium was placed in the dry incubator, covered with mineral oil, we observed a higher osmolality than the covered with paraffin oil.

**Conclusions:**

TWe can conclude that humid incubator is better for maintaining osmolality and paraffin oil protect single step media from evaporation in dry incubator.

## INTRODUCTION

Improvements in embryo culture protocols have allowed significant increase in ART treatment outcomes. Several types of culture media, oils, disposable materials and incubation systems have been exhaustively tested in the last 40 years (Swain, 2019). Some essential variables to consider within the culture system that include pH, temperature, gas concentration, osmolality and air quality, must be controlled to improve embryo development (Eaton et al., 2012; Swain et al., 2012; Heitmann et al., 2015; Swain, 2019).

Osmolality is a measure of solute particles dissolved in a solution calculated by an osmometer and is a well-known cell stressor that can impact embryo development. Human embryo development occurs in culture media under an osmolality range of 255–295 mOsm/kg (Erstad, 2003; Richards et al., 2010). Some variables such as preparation time, droplet size, temperature and air flow can impact evaporation during culture dish preparation, that can change culture media osmolality (Swain et al., 2012).

Culture media used for in vitro human embryo development is composed by aminoacids, salts, proteins, ions, water and a source of energy (pyruvate and glucose). Several different commercial culture media are available for ART treatments that can be for sequential or single step media (Bolton et al., 2014; Morbeck et al., 2014). Sequential media are based on two different formulations: one for the cleavage stage period (day1-day3), and the other for blastocyst stage (day 4-day6) (Gardner & Lane, 1997). Single step media is formulated to allow embryo development from day 1 to day 6 (Biggers & Racowsky, 2002).

Also, different types of oil can be used to cover the culture media droplets and minimize evaporation and pH fluctuations. Regardless the type of oil used, evaporation will occur. Therefore, the type of oil used, directly affects the speed of evaporation and heavy oils seems to provide less evaporation compared to a lighter oil (Kovačič, 2012; Swain, 2018). Paraffin and mineral oil are known to have different chemical properties, as mineral oil is lighter than paraffin (Otsuki et al., 2007). As mineral oil contains more unsaturated bonds than paraffin oil, it is more sensitive to photooxidation and peroxidation that is more detrimental to fertilization and embryo development (Elder et al., 2015). Some studies showed that use of paraffin oil results in less evaporation and higher incidence of good quality embryos (Sifer et al., 2009; Yumoto et al., 2018).

Additionally, the type of incubator, i.e., humid or dry, may also affect embryo development as it can interfere with contamination and evaporation rate of the culture media (Mori et al., 2010; Swain, 2014). Moreover, incubator oxygen level is crucial for better results in IVF cycles (Catt & Henman, 2000) as low oxygen concentration may enhance blastocyst development (Gardner & Lane, 1997; Bontekoe *et al*., 2012; Kovačič, 2012). Incubators with 5% of oxygen resulted in better results should be of reducing oxidation levels and maintaining the integrity of the amino acids in culture media (Biggers et al., 2004; Tarahomi et al., 2019).

Therefore, the aim of our study was to compare the impact of the type of culture media, type of oil used to cover the culture media and the type of incubator on the osmolality, during the embryo culture period.

## MATERIALS AND METHODS

We performed a prospective observational study between March and November 2019 to evaluate the osmolality of two different types of culture media covered by two different types of oil, placed in two types of incubator. As we did not use biological material an institutional review board approval was not necessary.

A total of 4 25µl droplets of single step medium (CSCM-C, Irvine Scientific, USA) and 4 25µl droplets of sequential medium (Sydney IVF Cleavage Medium, Cook, USA) were placed in 120 35×0 mm Petri dishes (Falcon, USA), both media in same dish. From those, 60 were covered by 3mL of paraffin oil (Ovoil, Vitrolife, Sweden) and 60 by 3mL of mineral oil (Light Mineral Oil, Irvine Scientific, USA). For each subgroup, 30 were placed in humid water jacket (Forma 4130, Thermo Scientific) and 30 in dry bench top (G185, K-Systems), both Tri-gas with 5% O_2_, 9.0% CO_2_, 86% N_2_, at the same temperature. All preparations were performed by the same person, using the same material, equipment, calibration, at the same temperature (23°C) and time of the day.

Weekly 20 Petri dishes were prepared, one by one, under a laminar flow hood at room temperature, using 25 microliter droplets of both culture media and covered with 3 mL of oil, mineral or paraffin. Osmolality (mOsm/kg) was measured on days 1, 3, 5 and 7 after preparation (one droplet per day) of each medium, covered with paraffin or mineral oil, placed in dry or humid incubator, using the same osmometer (Advanced Instruments 3320, USA), at the same time of the day and by the same embryologist. After collect the droplet for osmolality analysis dishes were replaced in same incubator until next measurement. Briefly, weekly we placed in dry incubator 5 dishes with 4 25µl droplets of CSCM-C + 4 25µl droplets of Cleavage Medium covered with mineral oil and 5 dishes with 4 25µl droplets of CSCM-C + 4 25µl droplets of Cleavage Medium covered with paraffin. In humid incubator we placed 5 dishes with 4 25µl droplets of CSCM-C + 4 25µl droplets of Cleavage Medium covered with mineral oil and 5 dishes with 4 25µl droplets of CSCM-C + 4 25µl droplets of Cleavage Medium covered with paraffin.

**Figure 1:**
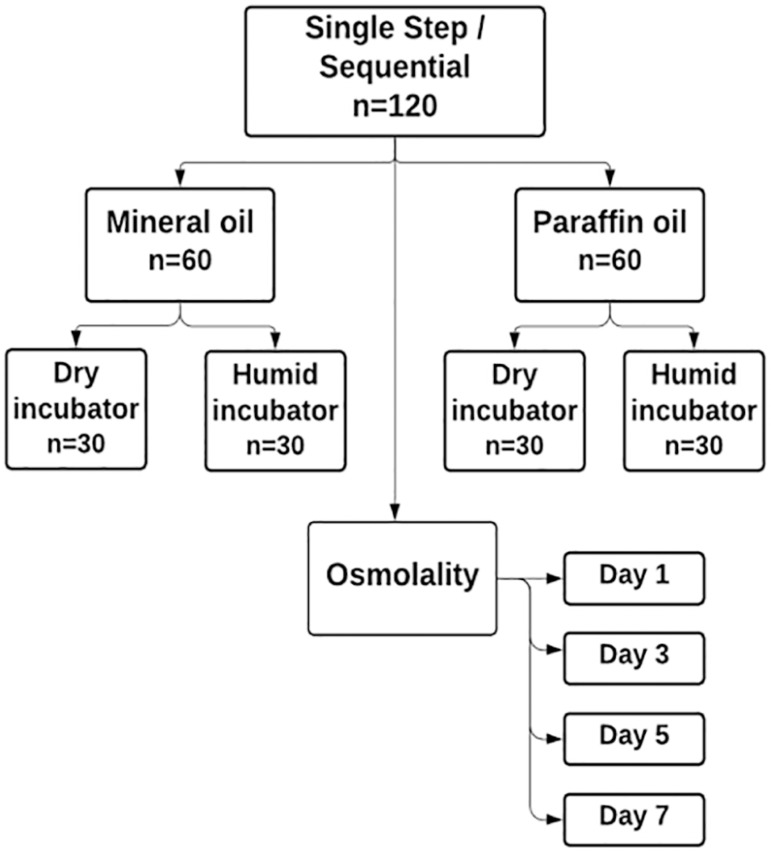
Diagram illustrating the experimental design: 120 dishes each one with 4 droplets of single step medium + 4 droplets of sequential medium (total of 960 droplets).

Therefore, in the end, we had eight groups: Group 1 (CSCM-C with mineral oil in dry incubator), Group 2 (CSCM-C with mineral oil in humid incubator), Group 3 (CSCM-C with paraffin oil in dry incubator), Group 4 (CSCM-C with paraffin oil in humid incubator), Group 5 (Cleavage with mineral oil in dry incubator), Group 6 (Cleavage with mineral oil in humid incubator), Group 7 (Cleavage with paraffin oil in dry incubator), Group 8 (Cleavage with paraffin oil in humid incubator) (Figure 1).

The sample size was calculated considering a 10% increase in osmolality result as the main variable. Thus, it was calculated that the analysis of 30 samples would provide a power test of 0.80 and alpha power of 0.05. So, for each day we measured 120 droplets of each culture media and used the mean±SD for analysis. Statistical analysis was performed using ANOVA test (SPSS 23.0, IBM) and post hoc Dunn-Sidák. Difference were considered significant at *p*<0.05.

## RESULTS

**Figure 2:**
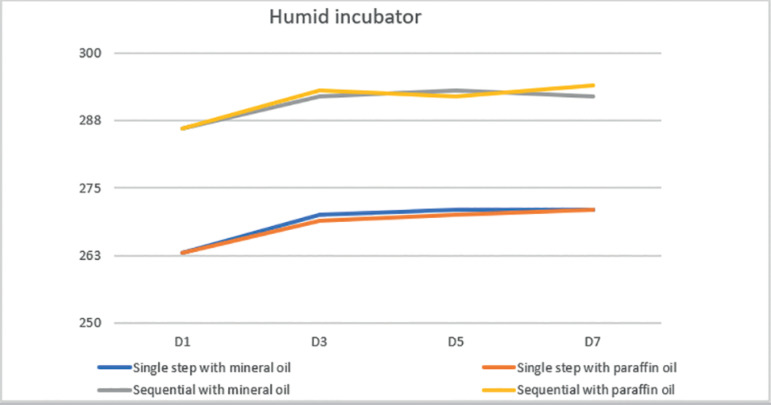
Mean osmolality of Single Step and Sequential media, covered with paraffin and mineral oil, in humid incubator on days 1, 3, 5 and 7.

**Figure 3:**
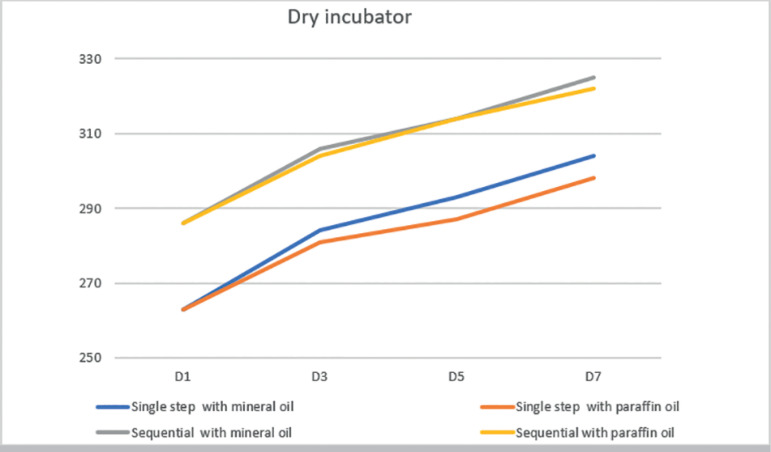
Mean osmolality of Single Step and Sequential media, covered with paraffin and mineral oil, in dry incubator on days 1, 3, 5 and 7.

A total of 960 droplets of culture media from 120 dishes, each one prepared with 4 droplets of CSCM-C and 4 droplets of Cleavage Medium, were evaluated for osmolality measurement, in four different moments. When we compared the osmolality of both culture media, we observed that single step medium (CSCM) had lower osmolality than sequential medium (Cleavage) in all periods, regardless of the type of oil or incubator (Figure 2 and Figure 3). Therefore, we performed a comparison on osmolality rate of change (slope) between different media.

When we compared the osmolality results on day 1 and day 3, in both culture media, covered with both type oils, we observed a significantly increase in osmolality regardless the type of incubator used, humid or dry.

When we analyzed the osmolality results of the single step culture medium, that were covered with both mineral and paraffin oil and placed in the dry incubator, we observed a significant increase in the results throughout the study time, i.e., D7>D5>D3 (*p*<0.001 for both oils) (Table 1). On the other hand, when we made de same comparison with the single step culture medium covered with both mineral and paraffin oil, placed in the humid incubator, the results were similar for all periods, i.e., D3=D5=D7 (Table 1).

**Table 1:** Results of Osmolality at the days 1, 3, 5 and 7 observed for Single Step Culture Medium, covered with paraffin and mineral oil, in Dry and Humid incubator.

	Single Step media													
	Mineral Oil							Paraffin Oil						
	Day 1	Day 3	p	Day 5	p	Day 7	p	Day 1	Day 3	p	Day 5	p	Day 7	p
Dry Incubator	263±1.22	284±3.8^a^	<0.001	293±3.54^b^	<0.001	304±3.59^c^	<0.001	263±1.22	281±3.21^d^	<0.001	287±3.13^e^	<0.001	298±2.60^f^	<0.001
Humid Incubator	263±1.22	270±2.67^a^	<0.001	271±3.74^b^	0.99	271±3.94^c^	0.89	263±1.22	269±2.67^d^	<0.001	270±3.24^e^	0.98	271±2.70^f^	0.51

ANOVA test - ^a^,^b^,^c^,^d^,^e^,^f^ *p*<0.001.

When we analyzed the osmolality results of the sequential medium, that were covered with both mineral and paraffin oil and placed in the dry incubator, we observed a significant increase in the results throughout the study time, i.e., D7>D5>D3 (*p*<0.001 for both oils) (Table 2). On the other hand, when we made de same comparison with sequential medium covered with both mineral and paraffin oil, placed in the humid incubator, the results were similar for all periods, i.e., D3=D5=D7 (Table 2).

**Table 2:** Results of Osmolality at the days 1, 3, 5 and 7 observed for Sequential Medium, covered with paraffin and mineral oil, in Dry and Humid incubator.

	Sequential medium													
	Mineral Oil							Paraffin Oil						
	Day 1	Day 3	p	Day 5	p	Day 7	p	Day 1	Day 3	p	Day 5	p	Day 7	p
Dry Incubator	286±2.40	306±3.50^a^	<0.001	314±4.42^b^	<0.001	325±3.74^c^	<0.001	286±2.40	304±3.75^d^	<0.001	314±4.18^e^	<0.001	322±2.88^f^	<0.001
Humid Incubator	286±2.40	292±3.32^a^	<0.001	293±2.58^b^	0.56	292±3.00^c^	0.99	286±2.40	293±4.24^d^	<0.001	292±3.43^e^	0.72	294±3.27^f^	0.41

ANOVA test - ^a^,^b^,^c^,^d^,^e^,^f^ *p*<0.001.

When we compared the impact of the different incubators on the culture media osmolality results, we observed that for both culture media, osmolality results were significantly lower in the humid incubators, in all periods, when droplets were covered with both oils (Table 1 and Table 2).

The impact of the different types of oil on the osmolality results was also evaluated and we observed that when the culture media was placed in the humid incubator, minimal variation was detected, in both culture media and in both oils. However, when single step culture medium was placed in the dry incubator, covered with mineral oil, we observed a higher osmolality than the covered with paraffin oil (Table 3). For sequential media, a significantly higher osmolality was observed only at day 7 in dry incubator, when covered with mineral oil (Table 4).

**Table 3:** Results of Osmolality at the days 1, 3, 5 and 7 observed for Single Step Culture Medium, covered with mineral and paraffin oil, in Dry and Humid incubator.

Single Step Medium	Dry incubator			Humid incubator		
	Mineral Oil	Paraffin Oil	p	Mineral Oil	Paraffin Oil	p
Day 1	263±1.22	263±1.22	1	263±1.22	263±1.22	1
Day 3	284±3.84	281±3.21	<0.001	270±2.67	269±2.67	0.39
Day 5	293±3.54	287±3.13	<0.001	271±3.74	270±3.24	0.98
Day 7	304±3.59	298±2.60	<0.001	271±3.94	271±2.70	0.99

ANOVA Test

**Table 4:** Results of Osmolality at the days 1, 3, 5 and 7 observed for Sequential Medium, covered with mineral and paraffin oil, in Dry and Humid incubator.

Sequential Medium	Dry incubator			Humid incubator		
	Mineral Oil	Paraffin Oil	p	Mineral Oil	Paraffin Oil	p
Day	286±2.4	286±2.4	1	286±2.4	286±2.4	1
Day 3	306±3.5	304±3.75	0.21	292±3.32	293±4.24	0.76
Day 5	314±4.42	314±4.18	1	293±2.58	292±3.43	0.91
Day 7	325±3.74	322±2.88	0.003	292±3.00	294±3.27	0.62

ANOVA Test

## DISCUSSION

Our study demonstrated that humid incubator is better than dry incubator for maintaining the osmolality of culture media, regardless of the type of culture media, as in the humid incubator, the osmolality remained almost unaltered after 7 days. Swain et al. (2016) and Yumoto et al. (2019) also observed an increase of the osmolality in culture media in dry incubator. This fact can be explained as the absence of humidity causes a higher evaporation rate and is independent of the type of oil used.

The mean osmolality, observed on day 1, of single step medium was 263 mOsm and the mean initial osmolality of sequential medium was 286 mOsm. According to Irvine Scientific osmolality ranges to 260-270 mOsm/ kg for CSCM-C and ranges to 285-295 mOsm/kg for Cook Cleavage Medium. Swain (2019) observed that an osmolality range of 255–265 mOsm/kg was ideal for single step media, used in an uninterrupted culture system, considering evaporation that occur in embryo culture. Also, osmolality values higher than 300 mOsm, are deleterious for embryo development. Thus, the osmolality obtained initially was in accordance with what was previously described as being optimal.

Osmolality changes may negatively affect embryos mitosis rates over the days in culture, as well as aneuploidies rates (Swain, 2019). Previous studies showed that the osmotic stress could disturbs the meiotic spindle in oocytes. Similar effects may occur in the embryo meiotic spindle, causing aneuploidies or mosaicism (Mullen et al., 2004). Lack of humidity causes a higher evaporation rate of the culture media and could be an additional source of stress to embryos. Changes in pH were also related to osmolality changes as pH depends on amount of water, salts, aminoacids, proteins, buffers and the incubator CO2 levels. Since the osmolality is compromised water evaporation, pH is also compromised (Swain, 2018; Gardner & Kelley, 2017). Other studies have also demonstrated the clinical relevance of the humidity in human embryo development. Fawzy et al. (2017) described lower clinical pregnancy rate when embryos were cultured in dry incubators when compared to those cultured in humid incubators. Del Gallego et al. (2018) observed a higher blastocyst formation after culture in humid conditions when compared to dry conditions.

In addition, incubator oxygen level is crucial for better results in IVF cycles (Catt & Henman, 2000). Low oxygen concentration may enhance blastocyst development (Gardner & Lane, 1997; Kovačič, 2012; Bontekoe et al., 2012). Guo et al. (2014) had significantly higher blastocyst rates when embryos were cultivated in incubators with 5% oxygen compared to the group with 20% of oxygen. Furthermore, incubators with 5% of oxygen obtained better results by reducing oxidation levels and maintain the integrity of the amino acids in culture media (Biggers et al., 2004; Tarahomi et al., 2019). Therefore, in our study we used only 5% oxygen incubators, dry or humid.

Oil is commonly used in embryo culture to minimize evaporation, fluctuations on pH and temperature of media (Otsuki et al., 2009; Labied et al., 2019). The type of oil used, directly affects the speed of evaporation: heavy oils provide less evaporation compared to a lighter oil (Swain, 2018). Our study showed that in dry incubator paraffin oil provided a greater protection against the evaporation to single step media on day 3, 5 and 7, which is essential for a successfully uninterrupted culture system. No significant difference was observed in sequential media on days 3 and 5. On day 7, the paraffin oil was better to maintain osmolality level. However, these media must be replaced by a proper media on day 3 of development. We measured osmolality of sequential media until day 7 as control to single step media. Swain (2018) also showed that heavier oil, provided a greater protection against the evaporation of media, if compared to the lighter oil in dry incubator. Moreover, it is important to note that osmolality had a significantly increased from day 1 until day 3 in both media, covered with both types of oils regardless the type if incubator used, probably due to media equilibration that occurs inside the incubators.

Oil composition is also an important factor that can affect IVF outcomes. Mineral oil is more likely to suffer photooxidation and peroxidation due unsatureted bonds which can lead to worse fertilization and embryo development (Otsuki et al., 2007). Sifer et al. (2009) compared commercial oils used in human embryo culture and showed that Ovoil promoted more top-quality embryos on day 3 than other mineral oil in humid conditions. Martinez et al. (2017) demonstrated that mineral oil affected negatively the cleavage rate of the embryo and the speed of the blastocyst formation, if compared with paraffin oil, probably due osmolality and pH changes. Nevertheless, Labied et al. (2019) analyzed embryos from dishes covered with paraffin and mineral oil and no diferences were observed in top quality embryos (41.7% × 41.2%), utilization rates (92.2% × 92.0%) and livebirth rates (26.9% × 26.2%). However, in this study they do not describe the type of incubator, humid or dry, used for embryo culture.

The type of culture medium might also have an impact on quality of the embryos generated from assisted reproduction techniques (Mantikou et al., 2013; Sepúlveda et al., 2009). Moreover, optimal performance of culture media depends on oil and incubator type used (Sifer et al., 2009). Nonetheless there is no consensus regarding of which culture media provides a higher livebirth rate (Sfontouris et al., 2016; Dieamant et al., 2017). Therefore, we performed all analysis using two different culture media, single step and sequential, in order to evaluate the possible effect of the different incubators and oils for both types. Our results demonstrated that, although single step media have lower osmolality, the variation observed through the study time was similar to the observed with the sequential medium

It is of fundamental importance how the dishes were prepared as Swain et al. (2012) observed an increase in osmolality when the temperature was 37°C. They also reported that 10µl droplets of culture medium have higher osmolality when compared to 20 or 40µl droplets. As all dishes were prepared by the same person, using the same material, equipment, calibration and temperature, at the same time of the day and the same volume of culture medium and oil, we did avoid any preparation bias that could interfere with the results.

In conclusion, our study demonstrated that humid incubator is better for maintaining osmolality and paraffin oil protect single step media from evaporation in dry incubator, which can improve continuous and undisturbed embryonic culture.
